# The Importance of Awaiting Biopsy Results in Solitary Pathological Proximal Femoral Fractures: Do We Need to Biopsy Solitary Pathological Fractures?

**DOI:** 10.1245/s10434-023-13931-4

**Published:** 2023-07-28

**Authors:** Floortje G. M. Verspoor, Gerjon Hannink, Michael Parry, Lee Jeys, Jonathan D. Stevenson

**Affiliations:** 1 Department of Oncology, Royal Orthopaedic Hospital, Birmingham, UK; 2 Amsterdam UMC, Department of Orthopaedic Surgery, University of Amsterdam, Amsterdam Movement Sciences, Amsterdam, The Netherlands; 3 Department of Operating Rooms, Radboud University Medical Center, Nijmegen, The Netherlands; 4 Aston University, Birmingham, UK

## Abstract

**Background:**

The optimal surgical treatment for patients presenting with (impending and complete) pathological proximal femoral fractures is predicated on prognosis. Guidelines recommend a preoperative biopsy to exclude sarcomas, however no evidence confirms a benefit.

**Objective:**

This study aimed to describe the diagnostic accuracy, morbidity and sarcoma incidence of biopsy results in these patients.

**Material and Methods:**

All patients (*n* = 153) presenting with pathological proximal femoral fractures between 2000 and 2019 were retrospectively evaluated. Patients after inadvertent surgery (*n* = 25) were excluded. Descriptive statistics were used to evaluate the accuracy and morbidity of diagnostic biopsies.

**Results:**

Of 112/128 patients who underwent biopsy, nine (8%) biopsies were unreliable either due to being inconclusive (*n* = 5) or because the diagnosis changed after resection (*n* = 4). Of impending fractures, 32% fractured following needle core biopsy. Median time from diagnosis to surgery was 30 days (interquartile range 21–46). The overall biopsy positive predictive value (PPV) to differentiate between sarcoma and non-sarcoma was 1.00 (95% confidence interval [CI] 0.88–1.00). In patients with a previous malignancy (*n* = 24), biopsy (*n* = 23) identified the diagnosis in 83% (PPV 0.91, 95% CI 0.71–0.99), of whom five (24%) patients had a new diagnosis. In patients without a history of cancer (*n* = 61), final diagnosis included carcinomas (*n* = 24, 39.3%), sarcomas (*n* = 24, 39.3%), or hematological malignancies (*n* = 13, 21.3%). Biopsy (*n* = 58) correctly identified the diagnosis in 66% of patients (PPV 0.80, 95% CI 0.67–0.90).

**Conclusion:**

This study confirms the importance of a preoperative biopsy in solitary pathological proximal femoral fractures due to the risk of sarcoma in patients with and without a history of cancer. However, biopsy delays the time to definite surgery, results can be inconclusive or false, and it risks completion of impending fractures.

Advances in the diagnosis and management of patients with non-sarcomatous malignancy have improved in recent decades, resulting in an increased prognosis, even in the presence of metastatic disease.^1–3^ Coupled with the advancing age of the population, the prevalence of metastatic disease increases.^4^ The proximal femur is one of the most common skeleton sites to develop metastatic disease and the most common site for pathological fractures.^5,6^

Pathological fractures are classically present with prodromal pain and an injury mechanism inconsistent with the injury. Impending fractures are often identified on radiographs investigating hip pain or during staging or surveillance following previous malignancy. Discriminating the underlying cause of pathological fractures is crucial to avoid inadvertent surgery which could compromise limb salvage and survival. The optimal surgical intervention for confirmed metastatic bone disease (MBD) is determined by an amalgamation of radiographic, patient, and tumoral factors.^7^ With the management of MBD, it must be remembered that (1) Pathological fractures will often not unite; (2) In certain cancer subtypes, excision of solitary deposits can result in improvement of overall survival (OS); and (3) The reconstruction must allow early weight-bearing, return of function, and outlive the patient’s lifetime.

Imperative to this decision making process is the underlying histological diagnosis. Inadvertent surgical intervention of sarcoma can be significantly detrimental to the patient’s prognosis and probability of achieving a cure. The indication for biopsy seems invariable to guide management in a patient presenting with a solitary lesion of bone, or with polyostotic disease of unknown origin.

This study aimed to identify the diagnostic accuracy, morbidity and sarcoma incidence of biopsy results for patients presenting with an (impending) pathological fracture of the proximal femur, in an attempt to answer the question, “is it mandatory to biopsy patients with a pathological or impending fracture of the proximal femur”?

## Material and Methods

**Fig. 1:**
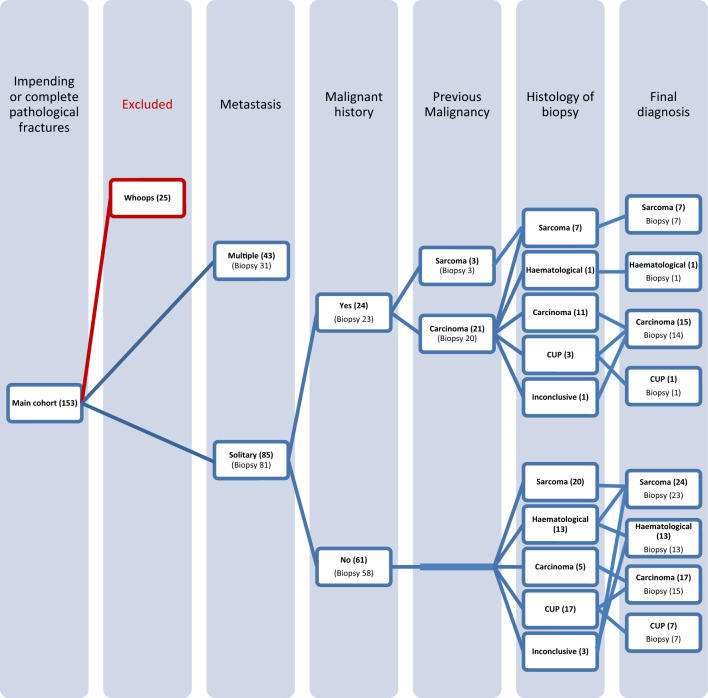
Flowchart of consecutive patients with impending and complete pathological proximal femoral fractures. Numbers shown in bold are the number of patients, while other numbers are the number of biopsies. Please note that four patients with solitary metastasis did not receive a biopsy before their final treatment, however they did have a final post-surgical resection diagnosis. In the ‘Final diagnosis’ column, the number of patients per histologic type is shown and whether these patients had a biopsy before further treatment. *CUP* carcinoma of unknown origin, *inconclusive* inconclusive biopsy results, *Whoops* inadvertent surgical procedure in which a surgeon is not aware of the diagnosis.

This study comprised a retrospective review of all patients presenting with suspected MBD to a single tertiary orthopedic oncology center between 2000 and 2019. Following local approval, patients were identified from a prospectively maintained database that records all patient episodes, including demographic, tumor and surgical factors. All patients were referred for investigation and management of an impending or pathological fracture of the proximal femur. Impending fractures were defined according to Sutter et al. as more than 50% of the width of the bone infiltrated on both the anteroposterior and lateral radiograph. Patients who had undergone inadvertent intervention at the referring center (*n* = 25) were excluded from the final study population. Therefore, the study population comprised 153 patients aged over 50 years with an impending or actual pathological fracture of the proximal femur (Fig. 1).

Primary outcomes were the diagnostic accuracy and morbidity of a needle core biopsy, including the risk of a pathological fracture in those with an impending fracture, and the delay in instigating treatment. Secondary outcomes included the incidence of primary sarcomas of bone, the incidence of a new second malignancy in patients with a previous malignancy, and the estimated survival of these patients with and without previous malignancy.

Demographics and collected variables can be found in Table 1. All patients underwent staging computed tomography (CT) scans of the thorax, abdomen and pelvis (CT-TAP), blood tests including myeloma screen and appropriate tumor markers (e.g. prostate-specific antigen [PSA]). Patients were classified as (1) Those with multiple metastases to bone and viscera; (2) Those with a solitary proximal femoral lesion (*n* = 85), (2a) with a history of cancer (*n* = 24, of which 23 were biopsied), and (2b) without a history of cancer (*n* = 61, of which 58 were biopsied). Patients with multiple metastases on staging (*n* = 43, of which 31 underwent biopsy), were excluded from the subsection analysis regarding secondary outcomes.

**Table 1:** Characteristics of patients with solitary pathological proximal femoral (impending) fracture

Variable		Malignant history [*n* = 24]	No malignant history [*n* = 61]
Median age at diagnosis, years (IQR)		64 (55–76)	66 (61–75)
Sex	Female	15 (62)	35 (57)
Side lesion	Left	15 (62)	32 (52)
Fracture	Actual	15 (62)	44 (72)
	Impending	9 (38)	17 (28)
Impending fracture fractured		2 (22)	7 (41)
Median time to fracture, days (IQR)		16 (10–21)	0.5 (0–19)
Fracture type	Neck of femur	5 (21)	17 (28)
	Pertrochanteric	5 (21)	13 (21)
	Subtrochanteric/diaphyseal	12 (50)	16 (26)
	Greater trochanter	1 (4)	3 (5)
	Lesser trochanter	0 (0)	4 (7)
	Unknown	1 (4)	8 (13)
Malignant history	Sarcoma	3 (12.5)	
	Carcinoma	21 (87.5)^a^	
Biopsy		23 (96)	58 (95)
Median time to biopsy, days (IQR)		17 (10–29)	14 (9–21)
Biopsy histology	Sarcoma	7 (29)	20 (35)
	Hematologic	1 (4)	13 (22)
	Carcinoma	11 (46)	5 (9)
	Carcinoma unknown	3 (13)	17 (29)
	Inconclusive	1 (4)	3 (5)
Diagnostic rate of biopsy		19 (83)	38 (66)
CT-TAP available		14 (58)	53 (91)
CT-TAP	Inconclusive	11 (79)	36 (68)
CUP^b^		2 (8)	15 (25)
Median time to treatment, days (IQR)		42 (27–56)	29 (21–41)
Procedure	Intramedullary nailing	2 (8)	1 (2)
	Endoprosthesis	17 (71)	49 (80)
	Plate	1 (4)	–
	Disarticulation hip	1 (4)	5 (8)
	Hindquarter amputation	–	3 (5)
	Non-surgical	1 (4)	2 (3)
	Unknown	2 (8)	1 (2)
Final diagnosis	Sarcoma	7 (29)	24 (39)
	Hematological	1 (4)	13 (21)
	Carcinoma	15 (63)	17 (28)
	CUP	1 (4)	7 (12)
Discrepancy	History and final diagnosis	5 (21)^c^	NA
	Biopsy and final diagnosis	–	4 (7)^d^
Follow-up	NED	–	11 (18)
	AWD	1 (4)	6 (10)
	DOD/DOOD	21/2 (96)	41/3 (72)
Survival from diagnosis, % (95% CI)	1 year	44 (23–62)	61 (47–73)
	2 years	26 (11–45)	49 (35–62)
	5 years	16 (4–34)	18 (7–32)

Data are expressed as *n* (%) unless otherwise specified

*IQR* interquartile range, *CT-TAP* computerized tomography scan of the thorax, abdomen and pelvis, *CUP* biopsy unknown and CT-TAP inconclusive, *NA* not applicable, *NED* no evidence of disease, *AWD* alive with disease, *DOD* death of disease, *DOOD* death of other disease, *CI* confidential interval

^a^History of carcinoma; breast (*n* = 8), bowel (*n* = 3), prostate (*n* = 2), head and neck (*n* = 2), bladder (*n* = 1), lung (*n* = 1), renal (*n* = 1), melanoma (*n* = 1), esophagus (*n* = 1), skin other (*n* = 1)

^b^CUP patients (*n* = 2) with a history of cancer resulted in carcinomas (one carcinoma of unknown origin) and CUP patients without a history of cancer resulted in 2 sarcomas, 1 hematologic malignancy and 12 carcinomas (7 carcinomas of unknown origin).

^c^Discrepancy between carcinoma history and final diagnosis (sarcoma [*n* = 4], hematologic malignancy [*n* = 1]).

^d^Discrepancy between biopsy and final diagnosis; inconclusive biopsies (*n* = 3) became a hematologic malignancy (*n* = 1) and two sarcomas at final diagnosis, one possible hematologic malignancy became a sarcoma at final diagnosis.

Biopsies (Jamshidi-needle, gauge 14, at least three macroscopically good quality samples) were performed under image guidance by fellowship-trained radiologists or orthopedic oncologists, and histopathology examination was performed by musculoskeletal pathologists with specialist training in bone sarcoma. No frozen sections are used in our center. All results were discussed in a specialist orthopedic oncology multidisciplinary meeting to plan the optimal management on an individualized basis.

### Statistical Analysis

Data were described using percentages for qualitative variables and medians with interquartile ranges (IQRs) for continuous variables. Descriptive statistics were used to summarize the data. The diagnostic accuracy of a biopsy was estimated and expressed as the positive predictive value (PPV) of the main histotypes (sarcoma, hematologic malignancy, or carcinoma) and, more specifically, if a biopsy can discriminate between carcinoma subtypes. Biopsy histology was taken as a test and final diagnosis (histology of specimen obtained during final surgery) as the reference standard. The fracture incidence of impending fractures was shown in percentages.

**Fig. 2:**
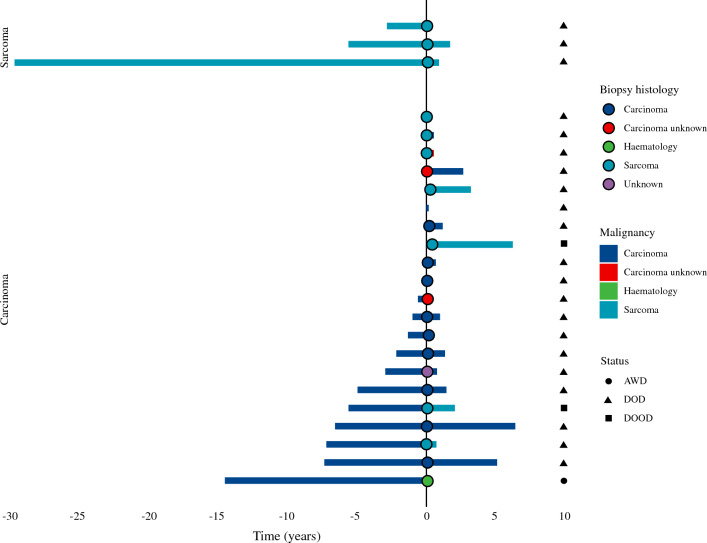
Swimmer plot with detailed information on biopsy and follow-up of patients with a solitary pathological proximal femoral fracture and a previous history of malignancy. The length of the bars indicates the time, in years, from first malignant diagnosis to the date of presentation with impending or pathological fracture (time point zero), followed by the length of follow-up after presentation. The colors indicate malignant history (bar to the left of the zero line), biopsy (circle), and final diagnosis (bar to the right of the zero line). *AWD* alive with disease, *DOD* death of disease, *DOOD* death of other disease

For patients with a malignant history, disease-free incidence, including malignant history, biopsy diagnosis, final diagnosis and follow-up status, was shown in a swimmer plot (Fig. 2).

Survival of patients was estimated using Kaplan–Meier analysis, with endpoint death of disease (DOD). Survival was calculated as the time from diagnosis to DOD or the end of study follow-up. Kaplan–Meier survival estimates were reported with their 95% confidence intervals (CIs). Log-rank tests were used to test for differences in survival between groups; patients with and without a malignant history, with an impending or actual pathological fracture, and by histotype (sarcoma, hematological malignancy, or carcinoma). All analyses were performed using R version 3.6.3 (The R Foundation for Statistical Computing, Vienna, Austria).

## Results

Overall, 153 patients presenting with impending and complete pathological fractures of the proximal femur between 2000 and 2019 were identified. After exclusion of patients who had undergone inadvertent procedures (*n* = 25), 128 patients remained, of whom 112 underwent biopsy and were used for further analysis (Fig. 1). Of the 16 patients where no biopsy was performed, the indication for no biopsy was due to the presence of advanced disease where management was purely palliative and biopsy would not have affected treatment. An overview of patient characteristics and oncological variables of patients with solitary lesions is presented in Table 1.

### Diagnostic Accuracy of Biopsy for Impending and Complete Pathological Fractures

Overall, 112 biopsies were performed that identified 30 (27%) sarcomas, 21 (19%) hematological malignancies, 55 (49%) carcinomas, and 5 (6%) unknown malignancies; 1 biopsy was non-malignant. In 25/55 (45%) carcinomas, biopsy failed to identify the primary site. After surgical resection, four patients had a different final diagnosis compared with their preoperative biopsy. Therefore, in 9 (8%) patients, biopsy resulted in an inconclusive or false-positive result. The PPV of a biopsy to differentiate between the major histology groups (carcinoma, sarcoma, hematological malignancy) was 0.96 (95% CI 0.91–0.99), while the PPV of a biopsy to differentiate between sarcoma and non-sarcoma in all patients was 1.00 (95% CI 0.88–1.00). The PPV decreased to 0.82 (95% CI 0.74–0.89) when discriminating carcinoma subtypes (determining the primary site of the metastasis).

Analysis of patients with solitary lesions and a previous malignancy (*n* = 24) showed that all but one case underwent biopsy (*n* = 23). Histological diagnosis was achieved in 19 (83%) cases; 7 sarcomas, 1 hematological malignancy, and 11 carcinomas. Three (12%) biopsies reported a carcinoma of unknown origin and one (4%) biopsy was inconclusive. The PPV of a biopsy to differentiate between the major histology groups (carcinoma, sarcoma, hematological malignancy) and correspond with the final resection pathology was 1.00 (95% CI 0.85–1.00). The PPV decreased to 0.91 (95% CI 0.71–0.99) when discriminating between carcinoma subtypes.

In patients with solitary tumors without a malignant history (*n* = 61), 58 biopsies were performed: 20 sarcomas, 13 hematological malignancies, and 22 carcinomas (of which 17 were of unknown origin); three (5%) biopsies were inconclusive. Histological diagnosis by biopsy was achieved in 38 (66%) cases. The PPV of a biopsy to differentiate between the major histology groups (carcinoma, sarcoma, hematological malignancy) was 0.98 (95% CI 0.90–1.00). This PPV decreased to 0.80 (95% CI 0.67–0.90) when discriminating between carcinoma subtypes (i.e., biopsy failed to identify the primary carcinoma site). Of three (5%) inconclusive biopsies, one was a hematological malignancy and two were sarcomas, confirmed after resection. One patient with a possible hematological malignancy at biopsy had sarcoma at post-resection histology, the only patient with a discrepancy between biopsy and final diagnosis.

### The Morbidity and Delay of Performing a Biopsy

For patients with a solitary lesion and a previous malignant history (*n* = 24), biopsy was completed in 23 patients at a median of 17 days (IQR 10–29) after presentation to the referring hospital. In patients with a solitary lesion without a malignant history (*n* = 61), 58 biopsies were performed after a median of 14 days (IQR 9–21) following presentation to the referring hospital.

Considering all biopsies for impending fractures (*n* = 38 of 112 biopsies), 12 (32%) patients suffered a complete pathological fracture during (*n* = 2) or after (*n* = 10) the biopsy, after a median 12 days (IQR 4–27). The median time from presentation to surgery was 30 days (IQR 21–45). Detailed information on fractured or impending solitary proximal femoral lesions is shown in Table 1.

Complications found after final fracture surgery, which risk might be increased by the delay of a biopsy, were urinary tract infection (*n* = 1), luxation (*n* = 1), bleeding (*n* = 1), temporary partial sciatic nerve palsy due to restoring length after fracture (*n* = 1), thrombosis (*n* = 4), and deep wound infection (*n* = 5) [Table 2].

**Table 2:** Treatments and complications after pathological proximal femoral (impending) fractures

Surgery location	No. of patients	Type of surgery	No. procedures	Complications	No. complications
Non-operative	9				
External surgery	10	Intramedullary nail	6	NA	
		Plate osteosynthesis	1	NA	
		THA	1	NA	
		Unknown	2	NA	
Internal surgery	109	Intramedullary nail	3		
		Plate	0		
		THA	12	Uninary tract infection	1
		Hemiarthroplasty	1		
		Hindquarter amputation	3		
		Hip disarticulation	6	Deep wound infection	2
		En bloc resection and EPR	84	Deep wound infection	3
				Thrombosis	4
				Implant failure	1
				Bleeding	1
				Neural^a^	1

After excluding WOOPS: *n* = 128

*External surgery* surgery at the local hospital, *internal surgery* surgery at the Royal Orthopedic Hospital, *THA* total hip arthroplasty, *EPR* proximal endoprosthetic femoral replacement, *NA* not available

^a^ Temporary sciatic nerve palsy due to restoring length after fracture

### Incidence of a New Second Malignancy with a History of Cancer

As shown in the swimmer plot (Fig. 2), three patients with a history of sarcoma (Ewing’s sarcoma [*n* = 1], soft tissue sarcomas [*n* = 2]) suffered a pathological fracture caused by relapsed sarcoma after a median of 5.6 years (IQR 4.3–17.7). In 21 patients with a prior carcinoma, 5 (24%) demonstrated a non-carcinoma malignancy on biopsy (sarcoma [*n* = 4], lymphoma [*n* = 1]). One patient with a history of breast cancer was diagnosed with lymphoma, three patients with a history of colorectal cancer developed chondrosarcoma, and one patient with a history of prostate cancer was diagnosed with radiation-induced osteoblastic osteosarcoma (consequence of previous cancer treatment). Median time interval from first carcinoma to presentation with a (pending) pathological fracture was 2.6 years (0.7–6.4).

Post-resection incidence of malignancies was 7 (29%) sarcomas, 1 (4%) hematological malignancy, and 16 (67%) carcinomas, of which one was a carcinoma of unknown primary (Fig. 2 and Table 1).

**Fig. 3:**
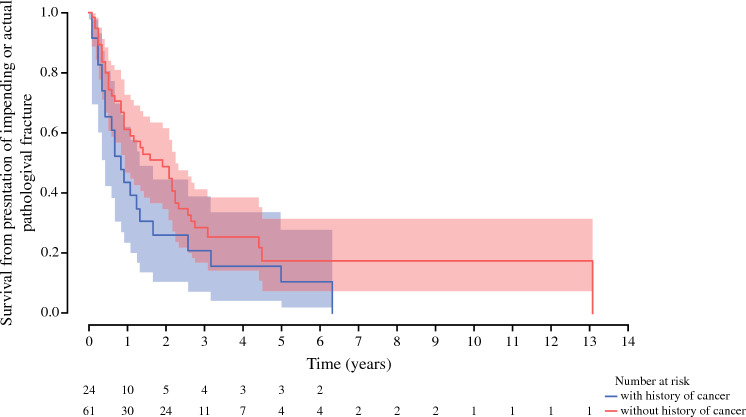
Kaplan–Meier curve for overall survival for patients with a solitary pathological proximal femoral (impending) fracture with (blue curve) and without (red curve) a history of cancer, showing no significant survival. The length of the bars indicates the time, in years, from first malignant diagnosis to the date of presentation with impending or pathological fracture (time point zero), followed by the length of follow-up after presentation. The colors indicate malignant history (bar to the left of the zero line), biopsy (circle), and final diagnosis (bar to the right of the zero line). *AWD* alive with disease, *DOD* death of disease, *DOOD* death of other disease

### Sarcoma incidence in Patients with Solitary Lesions Without a History of Cancer

In patients with solitary lesions without a history of cancer (*n* = 61), the post-resection histology showed 24 sarcomas, 13 hematological malignancies, and 24 carcinomas (7 of which were of unknown primary) [Fig. and Table 1].

### Survival in Patients with Solitary Lesions

Patients with a solitary lesion and a previous history of cancer (*n* = 24) had a median OS of 9 months (range 4–22), and 1-, 2- and 5-year survival rates from diagnosis of 44% (95% CI 23–62%), 26% (95% CI 11–45%), and 16% (95% CI 4–34%), respectively (Table 1, Fig. 3).

The median overall survival for patients with solitary lesions without a history of cancer (*n* = 61) was 12 months (range 4–31). The 1-, 2- and 5-year survival rates from presentation were 61% (95% CI 47–73%), 49% (95% CI 35–62%), and 18% (95% CI 7–32%), respectively. Seventeen patients had no evidence of disease (NED; *n* = 11) or were alive with disease (AWD; *n* = 6) at final follow-up (Table 1, Fig. 3). Those with a diagnosis of a hematological malignancy demonstrated the best overall survival based on histological diagnosis (1- and 2-year survival of 100 and 90% [95% CI 47–99%], respectively), and patients with carcinoma of unknown origin had the worst (1- and 2-year overall survival of 29% [95% CI 4–61%] and 14% [95% CI 1–47%], respectively).

## Discussion

To our knowledge, this study is the first to report outcomes of biopsy results in patients with solitary pathological proximal femoral fractures, with or without a history of cancer. We have confirmed that preoperative biopsy results reliably differentiate between histologic types (sarcoma, hematological malignancy, carcinoma) in patients with (96%) or without (95%) a history of cancer. However, biopsies were less helpful in discriminating between primary carcinoma sites of metastatic bone disease (agreement in 83 and 66%, respectively). In patients with a prior malignant history, almost one-quarter had a new second malignancy. In patients without a prior history of malignancy, over half of the patients, in whom MBD was considered, were diagnosed with a non-metastatic potentially curable malignancy (sarcoma or hematological).

The disadvantages of performing a biopsy are the risk of propagating an impending fracture into a complete fracture, and the morbidity associated with the delay necessitated by the process and interpretation of the biopsy. In patients with impending fractures, one-third did lead to completed pathological fracture following biopsy; however, this was often not at the time of biopsy but after a median period of 12 days post-biopsy, which may reflect the hazards of transferring patients to and from a diagnostic center and the delay of the pathway while awaiting the biopsy outcome before definitive treatment.

The time taken for completion of the pathway, which includes staging, referral, transfer, biopsy, and then definitive surgery, was approximately 1 month. While this timeframe appears quite long, this has improved significantly over time with the advent of improved image transfer, better communication between centers, and an appreciation of the importance of staging and prognostication before referral. Varady et al.^8^ showed no association of increased complications, including 30-day mortality or pulmonary complications, with delayed time to surgery after treatment of pathological hip fractures. This is in contrast to the standard management of conventional, non-pathological hip fractures, where surgery should be completed as soon as possible to prevent or reduce potential postoperative complications.^9–11^ The lack of an adverse effect associated with a surgical delay in patients with pathologic fractures could be explained by their younger average age compared with osteoporotic hip fractures.^12^ However, with delayed surgery, there is an increased risk of the extended postoperative length of stay, as a result of preoperative deterioration in functional and nutritional status.^8^

Taking into account the urgent need to establish a definitive diagnosis prior to a surgical intervention, the impact of delay caused by initiating a biopsy (inpatient stay, postoperative complications, or overall morbidity and mortality) should be minimized.

One in 12 patients had a discrepancy between biopsy and post-resection histology; in this cohort, the morbidity associated with biopsy and the delay in initiating treatment may be viewed as unnecessary because the biopsy did not influence the final histology. However, in 24% of patients with new solitary lesion with a history of previous malignancy, the biopsy differed from the previous malignancy, i.e. a second malignancy was diagnosed.

Metastatic patterns of disease from autopsy reports and cancer registries show prostate, breast, lung, and renal carcinoma are the main causes of bone metastases.^6^ However, bone metastases are often not specified by the primary site.^13,14^ Approximately 60% of impending or complete pathological fractures are located in the proximal femur causing disability and reduced quality of life.^15–20^ The present study describes the incidence of tumor types in patients with solitary proximal femoral (impending) fractures and highlights that a substantial proportion of solitary pathological fractures have a primary bone sarcoma or hematological malignancy, with and without a previous history of malignancy. This therefore justifies to biopsy these lesions to prevent inadvertent or inappropriate treatments in a significant number of patients.

The exact histological diagnosis is of clinical significance to the surgical management of pathological fractures by informing accurate prognosis.^21–24^ The representation of histotypes with intent-to-cure options in both patients with (>30%) and without (60%) malignant history was higher than expected. Zhang et. al. reported that over one-fifth of new bone lesions in patients with previous malignancy were second malignancies, of which 3.4% were newly discovered primary malignant bone tumors.^25^ Previous literature reported up to 18% of biopsy results identified new bone tumors unrelated to a previous malignancy.^26–29^ The present study shows a greater proportion of patients (24%) in whom a second primary malignancy was identified. This may reflect the nature of a tertiary referral center, a supraregional orthopedic oncology center responsible for the management of patients with primary malignant bone tumors. Referrals may be subject to a certain degree of prereferral selection bias and reflect an intention to exclude sarcoma. Lesions that are more likely to represent metastases from a carcinoma or hematological malignancy may not be referred to as often as those in which the radiology is less indicative or where a real diagnostic conundrum exists.

Surgical resection and durable reconstruction are advocated for patients with pathological fractures due to potentially curable (bone sarcoma, hematological malignancy) or solitary metastases (renal, breast, thyroid).^23^ The current study demonstrates that while reliable at distinguishing between the main tumor *types* (sarcoma, hematological or carcinoma), a biopsy was less effective at differentiating between histological subtypes (e.g., carcinomas). This is of relevance when considering surgical management, as prognosis predicates surgical management.^6^ This effect is even seen within subtypes of the same cancer entity, e.g. breast cancer,^30^ where prognosis differs between types (ductal, lobular) and hormone status (estrogen receptor [ER]/human epidermal growth factor receptor [HER]).^31,32^ The overall prognosis for newly diagnosed patients with breast, colorectal, and prostate cancers has globally increased, largely due to improvements in the medical management of the primary disease and of any subsequent metastatic disease.^7,33^ This will undoubtedly affect the incidence and prevalence of MBD in carcinoma patients, which makes the differentiation between subtypes all the more relevant.

Controversy exists on the impact of impending versus complete pathological fractures on outcomes of surgery. Some studies show impending fractures have better outcomes compared with surgery over actual pathological fractures.^34–37^ The current study found no significant survival difference between impending and actual proximal femoral fractures, justifying the inclusion of impending fractures in the analysis.

Limitations of the present study are excluding patients with inadvertent procedures at the expense of decreasing patient numbers, which aimed to reduce the heterogeneity of the cohort. Histology groups (sarcomas, carcinomas and hematological malignancies) are an oversimplification of cancer biology, but it is clinically relevant as these groups are managed by different multidisciplinary teams, and the use of neoadjuvant and adjuvant, non-surgical treatment modalities differs between these groups. Stratification by primary carcinoma was not included in this study, partly because biopsy in this context is so poor at discriminating between metastatic carcinomas. In clinical practice, this could lead to an overly optimistic or pessimistic survival prediction in more aggressive carcinoma subtypes, such as epidermal growth factor receptor-positive lung cancer or hormone-resistant prostate cancer.^38^ New developments in genomics, molecular analysis and the use of validated prognostic tools^39^ may offer targeted therapeutic regimens and improve the estimation of prognosis to aid surgical decision making.

## Conclusion

This study confirms the importance of a preoperative biopsy in solitary pathological impending and actual fractures of the proximal femur, both in patients with and without a history of malignancy. Solitary bone tumors should not be presumed to be MBD, due to the risk of inadvertent intervention of a solitary lesion that is in fact a sarcoma. The morbidity associated with biopsy, which includes completion of an impending fracture, delays in initiation of definitive treatment, and increased patient suffering, must be counterbalanced by the imperative need to exclude non-carcinoma diagnoses and thus prevent inadvertent surgery. Wherever possible, every effort must be made to expedite the transfer of information and the timely assessment of patients to ensure that wherever possible, treatment decisions are based on accurate information, including the results of biopsy where indicated.
